# Animal Models of Colorectal Cancer: From Spontaneous to Genetically Engineered Models and Their Applications

**DOI:** 10.3390/vetsci8040059

**Published:** 2021-04-05

**Authors:** Elisabete Nascimento-Gonçalves, Bruno A.L. Mendes, Rita Silva-Reis, Ana I. Faustino-Rocha, Adelina Gama, Paula A. Oliveira

**Affiliations:** 1 Center for the Research and Technology of Agro-Environmental and Biological Sciences (CITAB), University of Trás-os-Montes and Alto Douro (UTAD), 5000-801 Vila Real, Portugal; elisabete.nascimento.g@gmail.com (E.N.-G.); b.alex.mendes@hotmail.com (B.A.L.M.); ritareis96@hotmail.com (R.S.-R.); 2 Department of Zootechnics, School of Sciences and Technology, University of Évora, 7000-812 Évora, Portugal; 3 Department of Veterinary Sciences, University of Trás-os-Montes and Alto Douro (UTAD), 5000-801 Vila Real, Portugal; agama@utad.pt; 4 Animal and Veterinary Research Center (CECAV), University of Trás-os-Montes and Alto Douro (UTAD), 5000-801 Vila Real, Portugal

**Keywords:** spontaneous models, induced models, genetically engineered models

## Abstract

Colorectal cancer is one of the most common gastrointestinal malignancies in humans, affecting approximately 1.8 million people worldwide. This disease has a major social impact and high treatment costs. Animal models allow us to understand and follow the colon cancer progression; thus, in vivo studies are essential to improve and discover new ways of prevention and treatment. Dietary natural products have been under investigation for better and natural prevention, envisioning to show their potential. This manuscript intends to provide the readers a review of rodent colorectal cancer models available in the literature, highlighting their advantages and disadvantages, as well as their potential in the evaluation of several drugs and natural compounds’ effects on colorectal cancer.

## 1. Introduction

Worldwide, colorectal cancer is the third most common cancer in men and second in women [1]. Many risk factors have been considered for the development of colorectal cancer, such as the ingestion of processed meat, alcoholic drinks, body fatness, low intake of vegetables and fruits, smoking, and other concomitant diseases, such as inflammatory bowel disease (IBD), Crohn’s disease, and ulcerative colitis [2,3].

**Figure 1:**
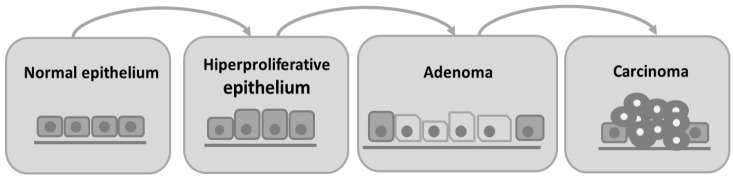
Schematic representation of adenoma–carcinoma multistep model. The normal cells of colon epithelium progress sequentially to a hyperproliferative epithelium, premalignant adenoma, and then carcinoma.

Colorectal cancer is characterized by the invasion of neoplastic epithelial cells below the muscularis mucosae of the colorectal wall [4]. Its evolution is slow and characterized by different stages. Progressive changes in the amount or activity of proteins that regulate cell proliferation, differentiation, and cell survival occur, leading to a disorder in cell replication that contributes to the development of proliferative lesions, such as adenoma [5]. Subsequently, the intestinal epithelium undergoes a malignant transformation to invasive carcinoma [4,5]. Besides adenomas, hyperplastic polyps, serrated adenomas, flat adenomas, and dysplastic lesions are also observed in the colon as other types of preneoplastic lesions [5]. In humans, colorectal cancer is histologically classified as an adenocarcinoma [4,6]. In Figure 1 we can observe the progression from normal intestinal epithelium to carcinoma.

About 97% of colorectal cancers are spontaneous, and the remaining are due to one of two autosomal dominant inherited diseases: hereditary non-polyposis colorectal cancer (HNPCC) and familial adenomatous polyposis (FAP) [4,5]. The genetic mechanisms of spontaneous CRC are present in the adenoma–carcinoma sequence. Carcinogenesis is initiated with inactivating mutations in the tumor suppressor adenomatous polyposis coli (APC) gene, followed by an accumulation of mutations in the genes K-RAS, PI3K, DCC, SMAD2, SMAD4, and lastly the mutation in the tumor suppressor gene TP53 that determines the progression from the non-invasive to the invasive CRC [7].

Laboratory rodents are commonly used as animal models in experimental research because they are easy and cheap to maintain, their physiology and genetics are well studied, and they are mammals like humans [8]. They allow us to understand and follow the progression of diseases, enable the discovery and development of new preventive strategies, which can be later used in clinical trials. An ideal animal model of human disease should be simple, not expensive, and mimic the disease in terms of morphology, biochemical alterations, and biological behavior [4,9]. Several works have reviewed the use of animal models of CRC [10]. However, this manuscript not only intends to augment the information on rodent models of CRC, highlighting their advantages and disadvantages, but also to review their applications and how they can be used to evaluate natural compounds, nutrition habits, and drugs.

## 2. Rat and Mouse Colon and Rectum: Anatomy and Histology

**Figure 2:**
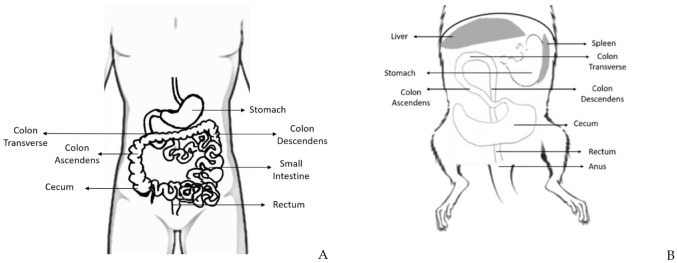
Schematic representation of some parts of the human (**A**) and rodent (**B**) digestive system, where is possible to observe the distinct portions of the large intestine: cecum, colon (ascendant, transverse, and descendent), rectum and anus, and its topographic anatomy. Human and rodent in supine position.

The rat and mouse intestine are similar to that of humans concerning development, structure, and functions [9]. The large intestine comprises the cecum, the colon, the rectum, and anus, and it is responsible for the absorption of water and salt from feces [11] (Figure 2).

The cecum is a curved blind sac responsible for bacterial fermentation and empties into the proximal/right colon. Even though the rodents’ colon and rectum represent a percentage of the total size of the large intestine similar to the humans, the cecum is much bigger in rats, which may be attributed to the high fiber content of their diet [12]. The colon continues toward the pyloric region of the stomach and has the same histological structure of the gastrointestinal tract: mucosa, submucosa, inner circular and outer longitudinal tunica muscularis, and serosa [1]. Despite histological similarities, rats and mice do not have adipose tissue in the submucosa, unlike humans who have it in abundance. The colon can be divided into ascending (it leads cranially to the thoracic cavity), transverse colon (from the left to the right side), and descending colon (on the right side of the abdominal cavity). The rodents’ middle and distal colon corresponds to the human left colon [13]. The rectum is relatively short and indistinct from the distal colon. The anorectal junction has no stratified columnar epithelium, and the anal canal is lined by keratinized stratified squamous epithelium [11].

## 3. Rodents as Models of Colorectal Cancer

Although there is no ideal animal model that replicates all human disease aspects, the rodents are accepted as good models to study colorectal carcinogenesis because of their physiological similarity with humans, reproducible tumor induction, and the possibility to study the disease biopathology and test strategies for cancer prevention and treatment [4].

**Figure 3:**
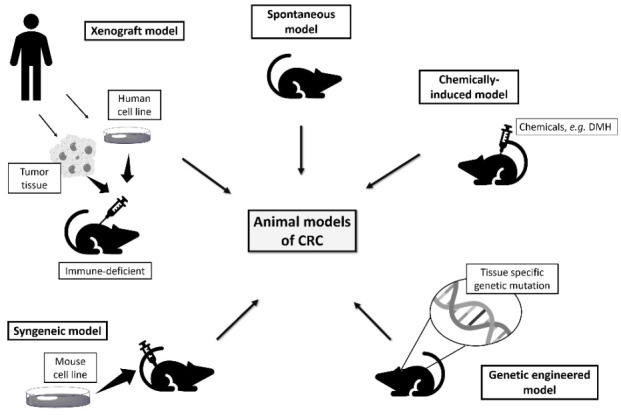
Rodent models available to study human colorectal cancer.

An ideal rodent model of colorectal cancer should develop carcinomas in the colon and rectum, with a high incidence in a short period, allow non-invasive monitoring of disease progression, and follow the histological and molecular characteristics of human colorectal cancer [8,13]. The models available to study colorectal cancer include spontaneous, induced, genetically engineered, xenograft, and syngeneic models (Figure 3).

### 3.1. Spontaneous Models

Spontaneous development of colorectal cancer in rats and mice is rare, although some cases were reported in the literature. In 1969 it was reported that C57BL mice developed adenomas in the colon [9], and in 1975 Miyamoto and colleagues showed that 30–40% of animals from the Wistar-Furth/Osaka strain developed adenocarcinomas [14]. More recently, in 2009, Newark and colleagues showed that C57BL/6J developed cancer in the large intestine with an incidence of 1% [15]. These models are not very used due to unpredictability and low reproducibility [4]. In 40% of the spontaneous rat models, the period of latency is approximately eight months [16,17].

### 3.2. Induced Models

Colorectal tumors can be induced in rodents through the administration of chemical carcinogens alone or in combination [5]. There are two types of chemical carcinogenic agents: direct and indirect. Direct carcinogens do not need to be metabolized to induce cancer, while the indirect agents are administered in their inactive form and only acquire carcinogenic activity when biotransformed and converted into their active form in the liver [5,6].

#### 3.2.1. Chemically Induced Models

In 1941, Lorenz and Stewart were the first to induce intestinal mouse tumors by feeding them with dibenzanthracene or methylcholanthrene [4], leading to the development of adenocarcinoma of the small intestine [4]. Later, in 1947, Lisco and colleagues induced carcinomas in the rat colon through feeding with radioactive yttrium [17]. Some years later, in 1963, Laquer and colleagues stated hydrazines are colonic carcinogens. Rats developed adenocarcinomas after feeding with a large quantity of cycad flour, which have hydrazine called cycasin, a form of methylazoxymethanol (MAM) [18,19].

Over the years, experimental research was conducted to discover chemical carcinogens specific for colorectal cancer such as 3,2′-dimethyl-4-aminobiphenyl (DMAB), alkylnitrosamines such as N-methyl-N-nitrosourea (MNU) and N-methyl-N-nitrosoguanidine (MNNG), 1,2-dimethylhydrazine (DMH), azoxymethane (AOM), and 2-amino-1-methyl-6-phenylimidazo (4,5-b) pyridine (PhIP) [20].

These carcinogens can be indirect-acting agents (DMAB, DMH, AOM, and PhiP), which need an enzymatic reaction to be converted into an active form, or direct-acting carcinogens (MNU and MNNG) that do not need biological catalysis [5].

##### 3,2′-Dimethyl-4-Aminobiphenyl (DMAB)

Walpole and colleagues, in 1952, described the first induction of intestine cancer in male rats by subcutaneous administration of DMAB dissolved in arachis oil, at a mean total dose of 2.8 g/kg, for 141 days [21]. After that, other researchers evaluated the carcinogenic potential of this compound [22]. Of these works, the one made by Reddy et al. [23] stands out; they showed that 30% of F344 rats fed with a low-fat diet and 75% of animals fed with a high-fat diet developed colon cancer after being injected with DMAB (50 mg/kg), once a week, for 20 weeks. DMAB forms carcinogenic DNA adducts through the N-hydroxylation by cytochrome P450, followed by O-acetylation and hydrolysis, reacting with DNA [23].

Nevertheless, this model has some disadvantages because multiple DMAB administrations are needed [23,24,25] and it has low specificity, leading to the development of tumors in various other tissues, such as salivary glands, mammary glands, urinary bladder, ear, and skin [6]. The studies performed using this model may be consulted in Table 1.

##### N-Methyl-N-Nitrosourea (MNU) and N-Methyl-N-Nitrosoguanidine (MNNG)

Since 1967, after discovering that oral administration of alkylnitrosamide induced adenocarcinomas in the glandular stomach in rats, other works were performed envisioning to address the carcinogenic potential of MNNG and MNU [4,13]. MNU and MNNG are direct DNA alkylating agents; they transfer a methyl group to nucleobases leading to the accumulation of genetic mutations [13]. Intra-rectal instillation of MNNG during 20 weeks at a dose of 1–3 mg/rat/week induced colon cancer in 100% of F344 rats [13,18,20]. Female ICR/Ha Swiss mice instilled with 0.3 mg of MNU intrarectally, three times a week for 10 weeks, developed tumors in the distal colon, rectum, and anus with an incidence of 78% [29]. The intrarectal administration allows a more selective induction in the distal colon and rectum, which is a huge advantage of this model. However, a precise technique is needed, and the quantification of drug volume is difficult [18,20]. In addition, the animals need to be kept in an inverted position for one minute after administration to prevent the return of the compound to the anus [19,20].

This model can be used to evaluate the therapeutic effects of several compounds on colorectal cancer development. More details about colorectal cancer studies using the MNU model to evaluate the influence of diet, drugs, and natural compounds can be consulted in Table 2.

##### 1,2-Dimethylhydrazine (DMH)

DMH is an alkylating agent that needs liver metabolic activation to become a carcinogen. Therefore, DMH is oxidized in the liver into azoxymethane and is then hydroxylated to form methylazoxymethanol (MAM). MAM is converted to formalin and methyldiazonium ion that are responsible for DNA, RNA, and protein alkylation [4,33].

The induction of colon cancer in rats with this compound was described for the first time in 1967 by Druckrey and colleagues, through its subcutaneous administration, at a dose of 21 mg/kg [20,34]. They showed that DMH cancer induction in the distal portion of the colon is histopathologically similar to humans [16,33]. These results were later confirmed by other authors [6,13,19].

DMH can be administered through different routes, including subcutaneous, intraperitoneal, oral, and intrarectal [4]. For example, a subcutaneous injection of 20 mg/kg DMH, once a week, for 20 weeks induces colonic adenomas in about 60% of male F344 rats [21]. Oral administration of 20 mg/kg showed a lower tumor incidence in male Wistar rats, depending on the nature of the diet [35]. Intrarectal administration of 250 mg/kg of DMH in Sprague-Dawley rats induced multiple colorectal adenocarcinomas with a latency period of 34 weeks [36]. Of the routes of administration presented, subcutaneous seems to be the one that leads to high incidence and consequently the most used in chemopreventive studies [4]. More information about other studies with this model may be consulted in Table 3.

**Figure 4:**
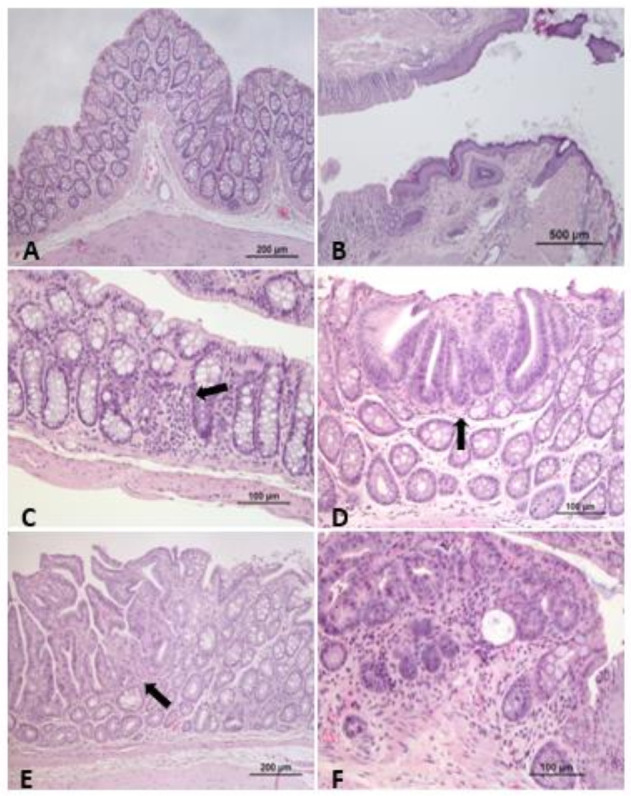
Representative photomicrographs of mouse/rat colon and rectum. (**A**) Rat colon with no alterations. (**B**) Mouse anorectal junction, with no alterations. (**C**) Mouse colon showing mild inflammatory infiltrate at the mucosa (DMH induction CRC model). (**D**) Rat colon with the presence of focal mild epithelial dysplasia (DMH induction CRC model). (**E**) Rat colon adenoma, characterized by a benign epithelial tubulopapillary neoplastic proliferation, non-invasive (DMH induction CRC model). (**F**) Mouse rectum, adenocarcinoma, characterized by a carcinomatous proliferation, associated with stromal invasion and inflammation (DMH induction CRC model). HE staining.

Although DMH-induced colon tumors in rodents are similar to human colon tumors [21], this model has disadvantages, e.g., multiple injections of DMH are necessary to induce tumors, it is characterized by at least six months of the latency period, and no hepatic metastases were observed until now (Figure 4C) [20].

##### Azoxymethane (AOM)

In 1970, Druckrey and colleagues showed for the first time the ability of azoxymethane (AOM) to induce intestinal tumors. Other works were then published confirming that AOM is a potent inducer of carcinomas of the large intestine in various strains of rats, such as F344, and mice, such as C57BL/6J and SWR/J, among others [21,37,38].

AOM is a metabolite of DMH that has been more frequently used in the induction of colon tumors than DMH, given some of its advantages over the original compound, such as its increased efficacy and greater chemical stability [37]. Like DMH, AOM is also an indirect carcinogenic compound, and it is activated in the liver by N-oxidation through cytochrome P450 2E1, producing metabolites such as methylazoxymethanol and methyl-diazoxide, which induce inflammation [39]. AOM seems to be a more effective carcinogen than DMH because it requires fewer reactions to be activated [6].

AOM induces rodent colon carcinogenesis when administered over 6–8 weeks via subcutaneous or intraperitoneal injection, with a latency period ranging from 20 to 30 weeks [13]. The distribution of tumors developed in the small intestine and colon (predominantly in the distal colon) is similar to that observed in the human colon [21,40]. Histological and histochemical properties of AOM-induced tumors are similar to those described in humans, being classified as adenomas and adenocarcinomas. Using this induction model, it was possible to identify metastases in lymph nodes and the liver similar to those described in humans [6,38].

Details concerning studies using AOM induction model to understand the influence of diet, drugs, or natural compounds in colorectal cancer can be consulted in Table 4.

**Table 3:** Studies using the DMH model to study different therapeutic approaches for colorectal cancer.

Animal Strain and Gender	Carcinogenic Administration Route	Drugs or Compounds Evaluated (Classification)	Dose/Treatment	Therapeutic Effects (Ref)
Wistar male rats	i.p. 40 mg/kg b.w. 2x/wk for 2 wks	Hyperbaric oxygen (HBO2)	HBO2 alone or DMH + HBO2; 15 daily 90 min HBO2 sessions every 24 h at 2.0 atm absolute pressure	HBO2 had a protective effect in colorectal cancer, demonstrated by the decrease in COX-2 [41]
	s.c. 40 mg/kg b.w. 2x/wk for 2wks	Astaxanthin	p.o. (15 mg/kg b.w.) 1 wk before and after DMH for 16 wks	Positive effects against colorectal cancer [42]
	s.c. 30 mg/kg 1x/week for 18 wks	Aspirin (a non-steroidal anti-inflammatory drug)	Gavage (0, 5, 30 or 60 mg/kg diet) daily for 18 wks	Reduced tumor incidence [43]
	s.c. 50 mg/kg b.w. 1 wk after diet supplemented and physical activity	Probiotic soy product and physical exercise	Gavage (3 mL/kg b.w./day fermented or unfermented soy products) and t.r. (60 min/day at 3–5% inclination at 355 m/min or 17–20 m/min) alone or in combination for 6 wks	No inhibition of colorectal cancer by the ingestion of fermented soy products or physical activity or by a combination of both [44]
	s.c. 40 mg/Kg b.w. for 8 wks	Epigallocatechin gallate (EGCG)	p.o. (50, 100 or 200 mg/Kg b.w.; once daily) for 8 wks	EGCG inhibited the formation of DMH-induced CRC by regulating key pathways, namely p53 and PI3K-Akt signaling pathways and I-kappaB kinase/NF-kappaB signal pathways, apoptosis signal pathways and MAPK cascades, involved in tumorigenesis [29]
Wistar female rats	s.c. 20 mg/kg b.w. for 20 wks	High fiber diet and aspirin	Exp1: gavage (10 or 30 mg/kg/day b.w aspirin) Exp.2: diet supplemented with high fiber (16% crude fiber) from the beginning and for 32 wks	Protective effects of high fiber diet and aspirin. The aspirin effect is dose-related [45]
	s.c. 20 mg/Kg b.w. for 5 wks	Methanolic extract of Muntingia calabura L. leaves (MEMC)	p.o. (100 or 200 mg/Kg b.w.) all days till the 15 week	MEMC offered a protective role against experimentally induced CRC via suppressing hyperproliferation and inflammation [46]
F344 male rats	Exp.1: s.c.20 mg/kg b.w. daily for 16 wks; Exp.2: s.c. 20 mg/kg b.w. daily for 12 wks	Cellulose, calcium and folic acid	p.o. (Exp.1: 10% cellulose for 30 wks; Exp.2: 250 or 500 mg/100 g diet calcium + 0 or 0.1 mg/100 g folic acid for 22 wks)	Protective effects of cellulose and reduced tumor number and multiplicity of calcium [47]
	i.p. 40 mg/kg b.w. once weekly for 4 wks	Adlay bran ethanol extract (ABE-Ea)	p.o. (8.64, 17.28 or 34.56 mg/day ABE-Ea) 1 wk before 1st DMH and for 9 or 18 wks	Inhibited preneoplastic lesions [48]
	i.p. 30 mg/kg weekly for 10 wks	Calcium and vitamin 3 in low or high-fat diet	p.o. (0.5 or 1%supplemental calcium and 1000 or 2000 IU/kg diet vitamin 3 in combination with a low-fat diet, 0.5% corn oil, or high-fat diet, 20% corn oil) 2 wks before DMH and for 20 wks	Preventive effects only in a high-fat diet [49]
	s.c. 100 mg/kg b.w. 2x/wk for 2 wks	Arabinoxylan-oligosaccharides (AXOS) (prebiotic)	p.o. (60 g/kg diet) 10 days before DMH and for 13 wks	Chemopreventive effect [50]
Sprague-Dawley male rats	Gavage 30 mg/kg	Aspirin (non-steroidal anti-inflammatory drug)	Exp.1: s.c.(100 mg/kg/day aspirin) 1 wk before and after DMH and for 1 wk; Exp.2: s.s. (50 mg/kg/day aspirin) 4 wks after DMH and for 36 wks)	Reduced tumor incidence when aspirin was administered 1 wk before or after DMH but no effect when administered 4 wks after [51]
	s.c. 30 mg/kg b.w. for 6 wks	etoricoxib (selective cycloxygenase-2 inhibitor) and diclofenac (a preferential cycloxygenase-2 inhibitor)	Gavage (0.6 mg/kg b.w. ectoricoxib and 8 mg/kg b.w. diclofenac) daily for 6 wks	Chemopreventive effect of both compounds [52]
	s.c. 20 mg/kg b.w. 2x/wk for 4 wks	Soy isoflavones	p.o. (1, 10, 50, 150 or 500 mg/kg diet) 1 wks after DMH and for 12 wks	Inhibited colorectal cancer in dose-independent manner [53]
	s.c. 20 mg/kg b.w. weekly from day 3 and for 12 wks	β-carotene (derived from carrots) sodium ascorbate (L-ascorbic acid) and cellulose	Diet supplemented (0.005% β-carotene or 0.02% sodium ascorbate or 1.5% cellulose) 12 wks before and for 28 wks	Only β-carotene showed an inhibitory effect of carcinogenesis [54]
	s.c. 20 mg/kg 1x/wk 13 wks	Wheat bran	Diet supplemented (fiber-free diet or 20% wheat bran supplement) for 31 wks	Increased colorectal carcinogenesis [55]
	s.c. 20 mg/kg b.w. 6x/wk	Milk and calcium	Diet supplemented (37 g/kg diet of milk and 40 mg/kg rat/day of calcium carbonate)	Protective activity by milk supplementation [56]
Sprague-Dawley male and female rats	s.c. 21 mg/kg 1x/week for 18 wks	Calcium	d.w.(3.2 g/L calcium lactate) daily from the start until 25–34 wks	Inhibited colorectal cancer [57]

b.w.: body weight; d.w.: drinking water; i.p.: intraperitoneal injection; p.o.: per os; s.c.: subcutaneous injection; t.r.: treadmill running; wk: week; wks: weeks.

**Table 4:** Studies using the AOM model to evaluate several therapeutic strategies for colorectal cancer.

Animal Strain and Gender	Carcinogenic Administration Route	Drugs or Compounds Evaluated (Classification)	Dose/Treatment	Therapeutic Effects (Ref)
F344 male rats	s.c. injections 15 mg/kg b.w./wk once a week for 2 wks	Ursodeoxycholic acid and cholic acid (bile acids)	p.o.(0.2% or 0.4% cholic acid, 0.2% or 0.4% ursodeoxycholic acid, 0.2% cholic acid + 0.2% ursodeoxycholic acid) for 30 wks	Higher dose of ursodeoxycholic acid reduced the incidence of colorectal tumors [58]
	s.c. 15 mg/kg bw once weekly for 2 wks	Celecoxib (a non-steroidal anti-inflammatory drug)	p.o. (500, 1000 or 1500 ppm) before exposure to AOM, during treatment, and until termination of the study at 52 wks	Chemopreventive activity in all tumor stages [59]
	s.c. 15 mg/kg b.w. 1x/wk for 2 wks	iNOS inhibitor L-N^6^ -(1-iminoethyl) lysine tetrazole-amide (SC-51), celocoxib (nonsteroidal anti-inflammatory)	p.o. (10, 30 or 100 ppm SC-51; 500 ppm celocoxib; 30 or 100 ppm SC-51 + 500 ppm celocoxib) for 8 wks	The combination of SC-51 with celocoxib was more effective in colorectal cancer prevention than the compounds alone [60]
	s.c. 15 mg/kg b.w. 1x/wk for 3 wks	Rebaudioside A, oleanolic acid, costunolide and soyasionin A2 (terpenoids), liquiritin (flavonoid), phyllodulcin and hydrangenol (isocumarins)	p.o. (200 ppm of each) for 5 wks	Costunolide is the most effective chemopreventive agent [61]
	s.c. 29.6 mg/kg b.w.	Piroxicam (a non-steroidal anti-inflammatory drug) and D, L-α-difluoromethylornithine (DFMO)	p.o. (25, 75 and 150 ppm piroxicam or 400, 1000 and 4000 ppm DFMO) 1 wk after AOM for 26 wks	A combination of piroxicam and DFMO was more effective in the inhibition of colorectal cancer than compounds alone [62]
	s.c. 15 mg/kg b.w. 1x/wk for 2 wks	Phenylethyl-3-methylcaffeate (PEMC)	p.o. (750 ppm) 2 wks before AOM for 52 wks	Inhibited colonic tumors [63]
	s.c. 15 mg/kg b.w. 1x/wk for 2 wks; start 2 wks after diet	Celocoxib (COX-2 inhibitor)	p.o. (1500 ppm) for 50 wks	Chemopreventive activity [64]
	s.c. 15 mg/kg b.w. at 7 and 8 wks of rat age	S-methylmethane thiosulfonate (S-MMTS) (isolate from cauliflower) and sulindac	p.o. (80 ppm S-MMTS, 160 ppm sulindac or 40 ppm S-MMTS + 160 ppm sulindac) 14 wks after AOM for	A combination of S-MMTS and sulindac was more effective in the inhibition of colorectal cancer than compounds alone [65]
	s.c. 15 mg/kg 1x/wk for 2 wks	Naproxen and NO-naproxen (nonspecific nonsteroidal anti-inflammatory drugs)	p.o. (200 or 400 ppm naproxen and 300 or 600 ppm nitric oxide-naproxen) 3 days after AOM for 8 wks	Chemopreventive effects [66]
	15 mg/kg i.p. 1x/wk for 2 wks	Lovastatin (statin) and exisulind (selective apoptotic antineoplastic drug)	p.o. (50 ppm lovastatin, 100, 250 or 1000 ppm exisulind alone or in combination with 50 ppm lovastatin) for for 4 wks	Chemopreventive effects of lovastatin but not exisulind [67]
	s.c. 15 mg/kg b.w. 1x/wk for 2 wks	CP-31398 (p53-modulating agent) and celocoxib (non-steroidal anti-inflammatory drug)	Diet supplemented (1, 150 or 300 ppm CP-31398, 300 ppm celecoxib or 1500 ppm CP-31398 + 300 ppm celecoxib) 2 wks after AOM and for 48 wks	A combination of compounds enhanced colorectal cancer chemopreventive efficacy [68]
	s.c. 15 mg/kg b.w. 1x/wk for 2 wks	Aspirin (a non-steroidal anti-inflammatory drug)	p.o. (0, 200 or 400 ppm) daily 2 wks before AOM and for 52 wks	Inhibited incidence and multiplicity of colorectal carcinomas [69]
	s.c. injection 15 mg/kg b.w. 1x/wk for 2 wks	Prebiotic germinated barley foodstuff (a mixture of insoluble protein and dietary fiber)	Diet supplemented with prebiotic germinated barley foodstuff for 4 wks	Anti-tumorigenicity activity [70]
	i.p. 15 mg/kg b.w.	Aspirin (a non-steroidal anti-inflammatory drug) and α-Difluoromethylornithine (DFMO) (ornithine decarboxylase inhibitor)	p.o. (Exp1.: 0, 200, 600 or 1800 mg/kg/diet of aspirin or 1000 mg/kg diet of DFMO; 8 days before 1st AOM; Exp.2: 200, 600, 1800 mg/kg/diet aspirin or 1000 or 3000 mg/kg/diet of DFMO or 1000 mg/kg/diet DFMO + 200 or 600 mg/kg/diet aspirin; 8 days before 1st) for 43 wks after last AOM	The combination of aspirin and DFMO after AOM reduced colorectal tumors [71]
	s.c. 15 mg/kg b.w 1x/wk for 2 wks	Vitamin D, acetylsalicylic acid (a non-steroid anti-inflammatory drug) and calcium	Diet supplemented (0, 2500, 5000 or 7500 ppm calcium; 0 or 300 ppm acetylsalicylic acid alone or combination with 0 or 0.02 µg/kg diet vitamin D) 20 days before AOM and for 18 wks	Increased incidence of tumors with high levels of calcium alone or in combination with vitamin D; Vitamin D with acetylsalicylic acid also increased tumor incidence [72]
	s.c. 8 mg/kg b.w./wk for 10 wks	Dietary wheat bran and dehydrated citrus fiber (in form of orange peel)	Diet supplemented (0 or 15% wheat bran or citrus fiber) for 20 wks	Reduced the risk of colorectal tumors [24]
	s.c. 15 mg/kg 1x/wk for 2 wks)	Tea extracts, Polyphenols and epigallocatechin gallate (EGCG)	d.w. (360 or 3600 ppm black and green tea extracts; 360 or 1800 ppm EGCG; 360 or 1800 black tea polyphenols and 360 or 3600 green tea polyphenols) at 6 wks and for 43 wks	No effect in tumor incidence [73]
	s.c. 15 mg/kg b.w. 1x/wk for 2 wks)	Aspirin, celecoxib, (cyclooxygenase-2 inhibitor), and atorvastatin (3-hydroxy-3-methylglutaryl CoA reductase inhibitors)	Diet supplemented (150 ppm atorvastatin, 600 pp celecoxib, 400 ppm aspirin, 100 ppm atorvastatin + 300 ppm celecoxib or 100 ppm atorvastatin + 200 ppm aspirin) one day after AOM and for 42 wks	Inhibited the incidence and multiplicity of colorectal carcinomas alone or in combination [74]
	s.c. 15 mg/kg b.w. 1x/wk for 2 wks	Grape seed extract (GSE)	Diet supplemented (0.25 or 0.5% (*w*/*w*) GSE) 1 wk before AOM, 4 wks last AOM or during all study and for 16 wks	Chemopreventive efficacy against early steps of colorectal carcinogenesis [75]
	s.c. 15 mg/kg b.w. 1x/wk for 2 wks)	Celecoxib (cyclooxygenase-2 inhibitor) in diets high in mixed lipids (HFML) or fish oil (HFFO)	Diet supplemented (0, 250, 500, or 1000 ppm celecoxib with HFML or HFFO diet) one day after AOM and for 26 wks	Preventive effect of low doses of celecoxib in HFFO diet [76]
F344 female rats	i.p. 20 mg/kg b.w.	Polyethylene-glycol (PEG) (non-fermented polymer)	Diet supplemented (3 g/kg b.w/day) 7 days after AOM and for 105 days	Chemopreventive effects [77]
	i.p. 20 mg/kg b.w.	Heme in food (in form of chicken, beef, black pudding)	Diet supplemented (600 g/kg diet chicken, beef and black pudding) 7 days after AOM and for 100 days	Increased colorectal carcinogenesis for all compounds [78]
	s.c. 8 mg/kg b.w./wk for 10 wks	Alfalfa, pectin and wheat bran	Diet supplemented (0 or 15% alfalfa, pectin and wheat bran) for 40 wks after 1st AOM	Inhibited colorectal tumor incidence, especially by pectin or wheat bran [79]
BALB-c female mice	i.p. 15 mg/kg1x/wk for 2 wks	Kefir (a probiotic fermented milk product)	p.o. (5 mL/kg b.w. fermented kefir milk) for 8 wks	Decreased and prevented the growth of colorectal tumors [80]
Sprague-Dawley male rats	s.c. 15 mg/kg 1x/wk for 2 wks, 28 days after diet supplementation	Amylose maize starch and butyrylated high-amylose maize starch	Diet supplemented (10% of high-amylose maize starch or 10% butyrylated alone or in combination) start at day 0 until euthanasia	The compound combination reduced the risk of developing colorectal cancer [81]
	i.p. 15 mg/kg 1x/wk for 4 wks	indomethacin and copper-indomethacin (non-steroidal anti-inflammatory drug)	i.p. (3.0 mg/kg indomethacin or 3.8 mg/kg copper-indomethacin) daily	Both compounds showed chemopreventive activity, but indomethacin was more effective [82]
	s.c. 15 mg/kg b.w. 1x/wk for 2 wks	R-Flurbiprofen (non-steroidal anti-inflammatory drug)	Gavage (30 mg/kg b.w./per day) 6 days a week, 1 wk before AOM and for 30 wks	Protective effects against colorectal cancer development [83]
	s.c. 15 mg/kg b.w. 1x/wk for 2 wks, at day 45 of rat’s life	Soy isoflavones	p.o. (0, 40 100 mg/kg diet) from birth, including pregnancy and lactation, until 26 wks of life. AOM at day 45	Lifetime exposure suppressed colon tumors growth [84]
	s.c. 15 mg/kg b.w. 1x/wk for 2 wks	Probiotic bacteria “*bifidobacterium lactis*” (*B. lactis*) and carbohydrate “resistant starch” (from a commercial source called Hi-maize 958 or Hi-maize S260)	Diet supplemented (100 g/kg/diet of Hi-maize 958 or Hi-miaze 260 and 1% lyophilized culture of *B. lactis*)	Protective effects by the combination of the two products [85]
	s.c. 15 mg/kg b.w. for three weekly doses	Xanthohumol (a polyphenol isolated from *Humulus lupulus L*.)	Gavage (5 mg/kg b.w.) every alternate day for 8 wks	Inhibited cell proliferation and induced apoptosis [86]
Wistar rats	i.p. 15 mg/kg	L-lysine, propolis, or gum arabic	Gavage water (150 mg/kg L.-lysine, 100 mg/5 mL/kg propolis or 5 mL/kg gum arabic) daily for 16 wks	Gum arabic and propolis reduced the total number of aberrant crypt foci, L-lysine neither protected against nor enhanced colorectal cancer [87]

b.w.: body weight; d.w.: drinking water; i.p.: intraperitoneal injection; p.o.: per os; s.c.: subcutaneous injection; wk: week; wks: weeks.

##### Azoxymethane (AOM) and Dextran-Sodium Sulfate Model (DSS)

Because colon cancer is associated with long-standing IBD, such as ulcerative colitis and Crohn’s disease, the risk of colorectal cancer development increases with the extent and duration of disease [19]. Chronic and repeated mucosal inflammation may result in tumors through several mechanisms, such as induction of genetic mutations, increased cryptal cell proliferation, changes in crypt cell metabolism, changes in bile acid, and alterations in the bacterial flora [5]. In 2003, Tanaka and colleagues developed a colitis-related mouse model of colorectal cancer initiated with AOM and promoted by dextran-sodium sulfate (DSS) [88]. DSS is an inflammatory compound that causes damages to the epithelial lining of the colon and induces colitis. Using this model, male Crj: Cd-1 (IRC) mice were intraperitoneally injected with AOM (10 mg/kg of body weight) and, one week later, received 2% of DSS in drinking water for seven days. Twenty weeks later, 88% of animals had colonic dysplasia, and the incidence of adenoma and adenocarcinoma was 100% [88]. This model allows the reduction in the number of AOM administrations, avoiding prolonged exposure to this compound, and still allows a reduction in the latency period. After this finding, other researchers have associated DSS with other compounds (PhIP and DMH), with tumor induction in a shorter period than the AOM/DSS model [19,89,90]. Concerning the AOM/DSS-induced model, different mice strains present diverse sensitivity; for example, the incidence and multiplicity of adenocarcinomas appear to be higher in the BALB/c mouse strain [91].

The AOM/DSS model mimetics human colorectal cancer pathogenesis, with a similar location (distal colon) and initiation by a polypoid growth. However, this model has a very low tendency to metastasize, which constitutes a limitation [92]. This model has been used in several chemopreventive studies of colitis-related colon carcinogenesis [88] (Table 5).

##### 2-Amino-1-Methyl-6-Phenylimidazo (4,5-b) Pyridine (PhIP)

The PhIP is a heterocyclic amine isolated from cooked fish and meat, which can be used to induce tumors in the colon, prostate, and mammary gland [93]. After administration, it is rapidly absorbed by the gastrointestinal tract and widely distributed through the body [19]. Then it is bio-transformed by the liver cytochrome P450 s, being converted in the amino group to a hydroxyamino group, which is then activated by forming esters with acetic acid, sulfuric acid, and proline. These esters are responsible to induce carcinogenic DNA adducts and genetic alterations leading to colorectal cancer [94]. The work performed by Ito and colleagues was the first to induce colon tumors in rats with this compound [95]. F344 rats from both sexes were fed with 400 ppm PhIP for up to 52 weeks and presented a high incidence of colon carcinomas [95]. PhIP did not induce colon cancer in mice, it just induced the formation of colonic aberrant crypt foci and lymphomas [4,9]. The mechanisms responsible for the non-induction of cancer in mice are not well understood [94]. This model has been used to evaluate the therapeutic effects of several compounds on colorectal cancer. Detailed information concerning mouse and rat models’ studies using PhIP induction can be consulted in Table 6.

**Table 5:** Colorectal cancer chemopreventive studies using the AOM/DSS model.

Animal Strain and Gender	Carcinogenic Administration Route	Drugs or Compounds Evaluated (Classification)	Dose/Treatment	Therapeutic Effects (Ref)
CF-1 male mice	s.c. AOM 10 mg/kg body wt + 1 wk later d.w. 1.2% DSS for 7 days	Aspirin (acetylsalicylic acid)	Diet supplemented (0.02% aspirin) 1 wk before AOM and for 20 wks	Suppressed inflammatory colitis symptoms and tumor multiplicity [96]
C57BL/6 male mice	i.p. AOM 10 mg/kg + 1 wk later d.w. 2% DSS for 1 wk	*Asther glehni Franchet et Sckmidt* (common Korean dietary edible herb)	p.o. (25 mg/kg/day) 1 wk after AOM + DSS and for 1 wk	Inhibited colitis-associated colon carcinogenesis [97]
	i.p. AOM 10 mg/kg b.w. + d.w. 2% DSS for 5 days	DA-6034 (7-Carboxymethyloxy-39,49,5-trimethoxyflavone) (synthetic derivative of flavonoid eupatilin)	Gavage (30 mg/kg) from day 7 to the end	Reduced the number of colon tumors [98]
	i.p. AOM 10 mg/kg b.w. + 5 days later d.w. 2.5% DSS followed by 14 days of normal water x 3 cycles	Pristimerin (a naturally triterpenoid)	i.p. (125 ng/kg) every 2 days for 80 days	Reduced the number and size of the tumors [99]
	i.p. AOM 10 mg/kg + 1 wk after d.w. 2% DSS for 1 wk	Chitooligosaccharides (oligomers that are depolymerized from chitosan)	i.g. (300 mg/kg) once a day and 6x/wk	Prevented colorectal cancer through regulating the gut microbiota and mycobiota [100]
C57BL/6 female mice	i.p. AOM 10 mg/kg + 1wk later d.w. 2% DSS for 1x/wk for 3 wks	Conjugated linoleic acid (CLA)	Diet supplemented (1% CLA) 3 wk before AOM + DSS and for 13 wks	Increased colorectal cancer [101]
	i.p. AOM 10 mg/kg b.w. + d.w. DSS 2.5% 1 wk after AOM for two cycle of 7 days	Licorice flavonoids (LFs) (Chinese herbal medicine)	Gavage (0, 50 or 100 mg/kg) once a day for 10 wks	Reduced tumorigenesis [102]
BALB/c female mice	i.p. AOM 10 mg/kg + 1 wk after d.w.DSS 2.5% 2.5% of 3 cycles of 1 wk	*Aloe vera* gel	p.o. (200 or 400 mg/kg/day) 1 wk before AOM and for 13 wks	Reduced the multiplicity of colorectal adenomas and adenocarcinomas [103]
BALB/c male mice	i.p. AOM 12.5 mg/kg + 1 wk after dDSS 2.5% in d.w. for 3 cycles of 5 days	*Triticum aestivum* sprouts ethanol extract (TAEE)	Gavage (100 or 200 mg/kg/day) for 40 days	Inhibited colon inflammation and neoplasm formation [104]
CD-1 (ICR) male mice	i.p. AOM 10 mg/kg b.w. + 1 wk after d.w. DSS 1.5% for 7 days	Zerumbone (tropical ginger sesquiterpene)	Diet supplemented (100, 250, or 500 ppm) for 17 wks	Zerumbone suppresses mouse colon carcinogenesis through mechanisms of growth, apoptosis, inflammation that are involved in carcinogenesis in the colon [105]
	i.p. AOM 10 mg/kg b.w. + 1 wk after d.w. DSS 1% for 7 days	Prenyloxycoumarins, auraptene and collinin nonsteroidal anti-inflammatory drugs)	Diet supplemented (0.01 and 0.05% of all compounds) 1 wk after DSS and for 17 wks	Chemopreventive activity [106]
	i.p. AOM 10 mg/kg b.w. + 1 wk after d.w. DSS 1% for 7 days	Ursodeoxycholic Acid (UDCA) and Sulfasalazine (anti-inflammatory agents)	Diet supplemented (0.016, 0.08 or 0.4% UDCA, 0.05% sulfasalazine or 0.5% UDCA + 0.05% sulfasalazine) 1 wk after DSS and for 20 wks	UDCA showed more suppressing effects on colorectal cancer [107]
	i.p. AOM 10 mg/kg b.w. + d.w. DSS 2.5% 1 wk after AOM and for 7 days	Dried açaí berry powder	Diet supplemented (2.5 or 5%) for 14 wks	Reduced the incidence of colorectal cancer [108]
	i.p. AOM (10 mg/kg b.w.) + d.w. DSS 1.5% 1 wk after AOM and for 7 days	Fucoxanthin (a xanthophyll present in marine brown algae)	Gavage (6 or 30 mg/kg)	Reduced the number of colorectal polyps [109]
CD-1 (ICR) female mice	i.p. AOM 10 mg/kg b.w. + 1 wk after d.w. DSS 2% for 7 days	Nimesulide (a cyclooxygenase-2 inhibitor), troglitazone and bezafibrate (ligands for peroxisome proliferator-activated receptors)	Diet supplemented (0.04% nimesulide, 0.05% troglitazone and 0.05% bezafibrate) 1 wk after DSS and for 14 wk	Suppressed development of colorectal cancer [110]
129SvJxC57BL6 male and female mice	i.p. AOM 12.5 mg/kg + 5 days later d.w. DSS 2% for 5 days followed by a 2-wk rest period and again 5 days of DSS	Chalcone lonchocarpin isolated from Lonchocarpus sericeus	i.p. (2.5 mg/mL) 4 wks after the last DSS cycle and for 4 days	Reduced tumor proliferation [111]

b.w.: body weight; d.w.: drinking water; i.g.: intragastrically; i.p.: intraperitoneal injection; p.o.: per os; s.c.: subcutaneous injection; wk: week; wks: weeks.

**Table 6:** Studies using PhIP model to study several strategies for colorectal cancer.

Animal Strain and Gender	Carcinogenic Administration Route	Drugs or Compounds Evaluated (Classification)	Dose/Treatment	Therapeutic Effects (Ref)
F344 male rats	p.o. 200 ppm for the first 20 wks	Tomato + broccoli powder in AIN93G diet	control, PhIP alone, PhIP + diet with 10% of tomato and broccoli powder for 20 wks and without PhIP for 32 wks	A diet rich in tomato + broccoli can reduce or prevent dietary carcinogens-induced cancer. Tomato + broccoli group reduced incidence and/or severity of cancer lesions [112]
	Gavage 75 mL/kg b.w. 5 times a week for 2 wks	Yogurt powder (milk fermented by *Lactobacillus delbrueckii* subsp. *Bulgaricus strain 2038* and *Streptococcus salivariu* subsp. *thermophilus* strain 1131)	Diet supplemented (10.4646% yogurt powder) 14 days before PhIP and for 14 days	Yogurt appears to have tumor-suppressing properties [113]
	Gavage daily 100 mg/kg b.w. for 2 wks	White tea, green tea, epigallocatechin-3-gallate (EGCG) and caffeine	d.w. (2% white tea, 2% green tea, 0.5 mg/mL EGCG or 9.5 mg/mL caffeine) 1 wk after last PhiP and for 16 wks	Inhibition of tumor initiation mostly by white tea, caffeine and EGCG [114]
	i.g. 100 mg/kg2x/wk for 10 wks	Nobiletin (5,6,7,8,3,4 -hexamethoxy flavone) (polymerthoxy-flavonoid extracted from citrus fruits)	Diet supplemented (0.05% nobiletin) for 50 wks	Chemopreventive activity of early carcinogenesis changes [115]
	Gavage 150 mg/kg for 5 alternate days	White tea	d.w. (2% wt/vol white tea) for 2 wks	Inhibition of preneoplastic lesion development [116]
	i.g. 200 mg/kg 2x/wk for 10 wks)	Fujiflavone (a commercial isoflavone supplement)	Diet supplemented (0.25% fujiflavone) for 50 wks	Preventive effects on colorectal cancer [117]
	Gavage 50 mg/kg b.w.	Clorophyllin (CHL) indole-3-carbinol (I3C)	p.o. (0.1% I3C and 0.1% CHL), before and during PhiP exposure or 1 wk after PhIP and for 16 wks	Protective effects for CHL and I3C on colorectal carcinogenesis [118]
F344 female rats	Diet supplemented 0.02%	caffeine, α-tocopherol (lipophilic antioxidant), and n-tritriacontane-16,18-dione (TTAD) (β-diketone derivative)	p.o. (0.1% caffeine, 0.5% α-tocopherol or 0.1% TTAD) for 54 wks	Increase the incidence of colorectal tumors by caffeine; α-tocopherol and TTAD had no effect on colorectal tumors [119]
Sprangue-Dawley male rats	Gavage 10 mg/kg b.w.	Chinese cabbage (*Brassica chinensis*)	p.o. (20% freeze-dried cabbage powder) 10 days before PhIP and for 20 h	Preventive effect on initiated colorectal tumors [120]

b.w.: body weight; d.w.: drinking water; h: hours; i.g.: intragastrically; p.o.: per os; wk: week; wks: weeks.

### 3.3. Genetically Engineered Models

Genetically engineered models allow the study of genetic predisposition to colorectal cancer development, and its interaction with environmental and modifying factors. These models mimic the genetic alterations of spontaneous and hereditary forms of colorectal cancer [9]. Through the study of hereditary colorectal syndromes, such as FAP and HNPCC, it was possible to discover the mutations under colonic carcinogenesis and replicate genetic lesions in mice and rats by developing genetically engineered models [9].

#### 3.3.1. Adenomatous Polyposis Mouse Models (APC)

It was demonstrated that human colorectal cancer is a multi-step genetic process and that the mutation of the APC gene occurs at the beginning of the carcinogenesis process. The APC gene is responsible for the regulation of β-catenin, cytoskeleton organization, cell cycle regulation, apoptosis, and cell adhesion. When mutated in the germline, this gene is associated with FAP [5,121].

Moser and colleagues discovered that C57BL/6 mice treated with ethylnitrosourea developed a mutation that predisposes to spontaneous development of intestinal cancer, naming the model as ApcMin mouse [122]. The Min mouse model is the only animal model of cancer that contains a single genetic alteration capable of producing a fully penetrating, consistent, and organ-specific tumor phenotype. The adenomas developed rapidly, with lesions identified within 60 days, and high tumor multiplicity. This model allows the study of multiple pathways impacting tumorigenesis and enables numerous entry points for basic or applied studies [10]. Over the years, this model has been tested, improved, characterized, and used to understand the role of the APC gene in colorectal cancer and also in chemopreventive studies [14] (Table 7).

Other mouse models with target genetic modifications at different locations on the APC gene have been generated, such as ApcMin/850, ApcΔ716, Apc1638N, Apc1638T, ApcΔ468, and ApcΔ474 [10], that allow the study of the colorectal cancer mechanisms, create models more similar to those changes found in humans, and test the role of specific regions in the APC gene on cancer development [9,10].

#### 3.3.2. F344-Pirc Rat Model

In 2007, Landgraf and colleagues developed a rat model carrying a knockout allele in the APC gene on an inbred F344/NTac genetic background rat [145]. To distinguish it from the APC mouse, they called this strain Pirc form (polyposis in the rat colon). The Pirc rats developed adenomas similar to those found in humans, showed the same progression to invasive carcinomas, and dependence on gender was observed, with males more prone to develop tumors in the intestinal tract than female rats [145]. In comparison to APC mouse models, this model takes some advantages due to the rat’s size, the facility of diagnostic imaging, colonic predisposition, and longevity [10].

#### 3.3.3. Hereditary Nonpolyposis Colon Cancer Mouse Models (HNPCC)

HNPCC is an inherited disease characterized by inactivated DNA mismatch repair genes, such as MLH1, MSH2, MSH6, and PMS2, that leads to the development of a variety of cancers, including colorectal cancer [15]. Mice with a targeted inactivating mutation in the mismatch repair genes are used to study these genes and evaluate their contribution to carcinogenesis [14,146]. Developed tumors are not specific to the colon-rectum; they occur in other organs such as the skin, lung, lymphatic system, stomach, and small intestine [15].

### 3.4. Xenograft and Syngeneic Models

**Figure 5:**
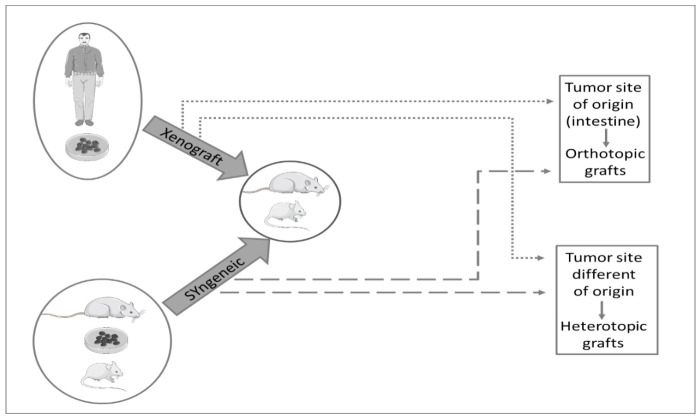
Schematic representation of xenograft and syngeneic models. In both models, the cells may be implanted in tumor site origin (orthotopic grafts) or a site different from tumor origin (heterotopic grafts).

Colorectal cancer cells or grafts of tissue can be implanted into animals to evaluate tumor development and to analyze the effects of several chemicals and natural compounds (Figure 5).

In xenograft models, human tumor cells or tumor fragments are implanted into immunocompromised animals. Syngeneic tumor models are characterized by the use of animal tumor cell lines obtained from chemically induced rodent colorectal cancer and are implanted into animals with the same genetic background as the cell line [13]. These models can be used, among others, to study the effects of treatment on colorectal cancer metastases [4,147,148].

The cells may be implanted subcutaneously, intrasplenically into the renal capsule (heterotopic models), or directly in the colon or rectum (orthotopic models) [13,14]. The subcutaneous inoculation (heterotopic model) is one of the most used methods due to the simplicity, easy access, and high tumor growth [4]; however, the tumor microenvironment is different from the colon [13], and the metastases do not develop [149]. Comparing with the subcutaneous heterotopic model, the intrasplenically and renal capsule heterotopic models and the orthotopic model are technically more advanced and more difficult to work with, requiring the animals’ anesthesia and use of imaging modalities (e.g., ultrasonography) to implant the cells specifically in the spleen, the renal capsule or colon, respectively [150].

Orthotopic implantation refers to cells or tumor fragments implanted in the tumor site of origin, i.e., colon or rectum [13]. These models allow replication of tumor invasion, vascular spread, mimic the progression to advanced colorectal cancer in humans, and metastasize to distal organs [12]. For example, MCA-38 cells were intramurally injected into the cecum of C5BL/6J mice, and 40–65% of them developed metastases eight weeks later [151]. In another study, CT26 cells were transanal rectal injected in Balb/c mice with uptake rates of 65%, but only 3.3% developed metastases [152].

These models can be used to evaluate some therapeutic drugs. For example, Tao and colleagues used a commercial human colon cancer cell line, HCT-116, to evaluate the anti-colorectal cancer activity of Weichang’an, a Chinese herbal medicine, with 5-fluorouracil. The cells were injected subcutaneously in male BALB/C mice axilla, and after tumor growth, they were transplanted into the cecum. The group concluded that the compounds evaluated inhibited both colon tumor growth and hepatic metastases [153].

## 4. Conclusions

Experimental data concerning dietary, drugs, and natural compounds’ effects on colon cancer models were reviewed in this work. Although several animal models are available to study colon cancer, there is no perfect model; all constitute an important tool to study human and animal colon carcinogenesis and to evaluate the potential effects of preventive and therapeutic strategies. 

Whereas the AOM/DSS model mimetics the pathogenesis observed in human colorectal cancer, others like genetically engineered models allow studying genetic predisposition to the development of this type of cancer. The model selection should consider the studies’ goals, the costs, and the advantages and disadvantages of each model, animal, strain, and gender.

Considering dietary patterns and natural products used as chemoprevention or chemotherapy, some like soy isoflavones, β-carotene, dried plums, fuji flavone, and Chinese cabbage showed an inhibitory effect on colorectal carcinogenesis, and adlay bran ethanol extract, grape seed extract, and pomegranate peel extract decreased the development of colonic premalignant lesions. However, groups that studied the effects of wheat bran and heme groups (in form of chicken, beef, black pudding) in the mice diet have concluded the opposite, observing a higher incidence of colorectal carcinogenesis.

In some cases, natural compounds, several drugs, and dietary patterns results are inconsistent and depend on multiple factors, and the best way to obtain better results is to select the most appropriate model and try to reduce most of the external factors. To achieve this goal, more research with controlled parameters is warranted. Moreover, ideally, the studies to evaluate the effects of natural compounds in CRC should not only evaluate the whole compound, but also each active substance in an isolated way. However, these studies imply the use of a higher number of animals, and consequently, higher costs for researchers, which may constitute a limitation.

## Figures and Tables

**Table 1:** Studies using the DMAB model to study different therapeutic approaches for colorectal cancer.

Animal Strain and Gender	Carcinogenic Administration Route	Drugs or Compounds Evaluated (Classification)	Dose/Treatment	Therapeutic Effects (Ref)
F344 male rats	s.c. 100 mg/kg b.w.	Copper-zinc (CU), manganese (Mn), and iron (Fe)	p.o. (0.8 or 5.1 µg CU/g diet; 0.6 or 17 µg Mn/g diet and 37 or 140 µg Fe/g diet) 3.5 wks before DMAB and for 8 wks	Increased neoplastic lesions by low doses of copper and manganese relative to iron [25]
	s.c. 100 mg/kg b.w. 1/wk for 2 wks	Selenium (nutritionally essential trace element)	p.o. (0, 0.1 or 2.0 mg selenium/kg diet as selenite, selenate or selenomethionine) 4 wks before DAMBP for 12 wks	Dietary administration of selenium in the form of the inorganic salts selenite and selenate reduced colon ACF [26]
	s.c. 100 mg/kg b.w.	Celecoxib (selective cyclooxygenase-2 inhibitor)	Diet supplemented (0, 500, 1000, or 1500 ppm celecoxib) 2 wks before DMABP and for 2 days	Chemopreventive effect for colorectal cancer in a dose-response manner [27]
	Gavage 50 or 5 mg/kg b.w. 1/wk for 4 wks	Acetaminophen	Diet supplemented (1000 ppm) 2 wks before DMAB and for 6 wks	Protective effect on the development of colorectal carcinogenesis [28]

ACF: aberrant crypt foci; b.w.: body weight; p.o.: per os; s.c.: subcutaneous injection; wk: week; wks: weeks.

**Table 2:** Studies using the MNU model to evaluate the effects of different therapeutic strategies for colorectal cancer.

Animal Strain and Gender	Carcinogenic Administration Route	Drugs or Compounds Evaluated (Classification)	Dose/Treatment	Therapeutic Effects [Ref]
F344/NSlc female rats	i.r. 2 mg 3x/wk for 3 wks	Ursodeoxycholic acid (UDCA) and 5-aminosalicylic acid (5-ASA) (non-steroid anti-inflammatory drug)	p.o. (0%, 0.11 or 0.02%) for 30 wks	Inhibited colorectal cancer development [29]
Sprague Dawley female rats	i.r. 10 mg/Kg 3x/wk for 4 wks	Omega 3 polyunsaturated fatty acid (Omega-3PUFA)	i.g. (2 g/kg) daily for 4 wks	Attenuated CRC by blocking PI3K/AKT/Bcl-2 [30]
F344 male rats	i.r. 2 mg/rat 3x/wk for 4 wks	4-[3,5-Bis(trimethylsilyl)benzamido]benzoic acid (TAC-101) (retinobenzoic acid derivative)	p.o. (0.8 or 8 mg/kg for 1 or 4 wks) for 20 wks	Inhibited colorectal cancer development [31]
	i.r. 4 mg on days 1 and 4	Calcium and cholic (bile acid)	d.t. (0.2% cholic acid or 1.6% calcium) for 28 wks	Increased colorectal tumor development by bile acid and no protective effect of calcium [32]

b.w.: body weight; d.t.: diet supplement. i.g.: intra-gastrically; i.r.: intra-rectal administration; p.o.: per os; wks: weeks.

**Table 7:** Studies using the APC^Min/+^ model to evaluate the effect of several compounds in colorectal cancer.

Animal Strain/Gender	Drugs or Compounds Evaluated (Classification)	Dose/Treatment	Therapeutic Effects (Ref)
Min mice/n.d.	α-phenyl-tert-butyl nitrone (PBN) and 4-hydroxyl-PBN (4-O-PBN) (nitrones)	d.w. (100 ng/kg/day PBN or 4-O-PBN) for 3–4 months	Anti-cancer activity of PBN more significant than 4-O-PBN [123]
	Bilberry (Vaccinium myrtillus), lingonberry (Vaccinium vitis-idaea), cloudberry (Rubus chamaemorus), cloudberry seeds or cloudberry pulp, or pure ellagic acid	p.o. (1564 mg/kg of each) for 10 wks.	Chemopreventive potential [124]
	Atorvastatin (hydroxy-3- methylglutaryl CoA reductase (HMGR) inhibitor) and celecoxib (cyclooxygenase-2 (COX-2) inhibitors)	p.o. (0 or 100 ppm atorvastatin or 300 celecoxib alone or in combination) for 80 days	Inhibited intestinal tumorigenesis by atorvastatin and increased chemopreventive activity in combination with celecoxib [125]
	Piroxicam (a non-steroidal anti-inflammatory drug)	p.o. (200 ppm piroxicam) for 100 or 200 days of rat age	Reduced tumor multiplicity [126]
	Annurca Apple polyphenol extract	d.w. (60 µmol/L) in combination with a western diet or balanced diet for 12 wks	Chemopreventive potential [127]
	Fermented brown rice and rice bran (FBRA)	Exp.1: diet supplemented (5 or 10% FBRA) for 20 wks; Exp2: diet supplemented (5 or 10% FBRA and 2% DSS in d.w. for 1 wk) for 12 wks; Exp3: diet supplemented (10% FBRA and 1.5% DSS in d.w. for 1 wk) for 7 wks	No effect on tumor development by FBRA alone but in combination with DSS suppressed the multiplicity of colon tumors [128]
	Sulforaphane (SFN) (isothiocyanate)	Diet supplemented (600 ppm SFN) for 1, 3, or 5 days	Chemopreventive potential [129]
	Bilirubin, bovine serum albumin (BSA) and sodium taurocholate (NaTC)	p.o (0.5 mM BSA alone or in combination with 0.25 mM bilirubin or 5 mM NaTC) for 8 wks	Reduced intestinal adenomas by NaTC [130]
	Metformin (biguanide derivative)	p.o. (250 mg/kg/day) for 10 wks	Chemopreventive potential [131]
	Silibinin	Gavage (750 mg/kg b.w.) for 5 days a wk for 13 wks	Chemopreventive potential [132]
Min mice/female and male	Aspirin	Diet supplemented (250 or 500 ppm) for 7 wks	Chemopreventive potential [133]
	Curcumin	Diet supplemented (2% curcumin) from 4 to 18 wks of age	Chemopreventive potential [134]
	Anthocyanin-rich tart cherry extract and sulindac (a nonsteroidal anti-inflammatory drug)	p.o. (0, 375, 750, 1500 or 3000 mg anthocyanin-rich tar cherry extract/kg if diet with 100 mg sulindac/kg diet) for 19 wks	The combination of both compounds had a more protective effect than compounds alone [135]
	Physical activity	t.r. (18 m/min, 60 min/day, 6 days/wk or voluntary wheel running) for 9 wks	Reduced number and size of intestinal polyps, dependent on exercise mode and gender [136]
	DMU-135 (3,4-Methylenedioxy-3,4,5 -trimethoxy chalcone) (anticancer prodrug)	Diet supplemented (0.2% w:w) from 4–18 wks	Chemopreventive activity [137]
	MCC-555 (peroxisome proliferator-activated receptor (PPAR) ligand)	Gavage (30 mg/kg/day 5 days/wk) for 4 wks	Suppressed activity [138]
	Soy isoflavones	Diet supplemented (low-isoflavone: 11.5 genistein, 2.3 daidzein and 2.3 mg of glycitein/kg diet, rich isoflavone diet: 280.6 genistein, 147.2 daidzein and 48.3 mg of glycitein/kg diet) for 107 days	No inhibition of colorectal tumor development [139]
Min mice/male	Orange peel extract (OPE)	Diet supplemented (0.25 or 0.5% OPE) for 9 wks	Inhibited colorectal tumorigenesis [140]
	Physical activity	t.r.(18 m/min, 60 min, 6 days/wk, 5% grade) for 9 wks	Reduced the overall tumor burden (size and number) [141]
	Guanidinoethyldisulfide (GED) [14,15,16,17], peroxynitrite decomposition catalyst, FP 15 and poly(ADPribose) synthetase (PARP) inhibitor, N-(6-oxo-5,6-dihydrophenanthridin- 2-yl)-N, N-dimethylacetamide hydrochloride (PJ 34) (specific inhibitors of inducible nitric oxide synthase)	Gavage (10 or 30 mg/kg/day GED, 1 or 3 mg/kg/day FP15 and 3 or 10 mg/kg/day PJ34) twice a day from 5 wks of age until 12 wks	Chemopreventive activity of all compounds [142]
Min mice/female	(–)-epigallocatechin-3-gallate (EGCG) and fish oil	Diet supplemented (0.16% EGCG alone or in combination with high-fat fish oil diet, 20% w:w) for 9 wks	Inhibited tumor multiplicity by a combination of low doses of EGCG and fish oil [143]
Apc1638N mice/male and female	Aspirin and α-amylase resistant starch (RS)	Diet supplemented (125 g/kg diet RS or 0.3 g/kg aspirin alone or in combination) from 6 wks	The combination of two compounds showed more preventive activity than compounds alone [144]

d.w.: drinking water; n.d.: no data; p.o.: per os; t.r.: treadmill running; wk: week; wks: weeks.

## Data Availability

Data sharing not applicable.
